# Efficient D-allulose synthesis under acidic conditions by auto-inducing expression of the tandem D-allulose 3-epimerase genes in *Bacillus subtilis*

**DOI:** 10.1186/s12934-022-01789-2

**Published:** 2022-04-19

**Authors:** Mengkai Hu, Yuxia Wei, Rongzhen Zhang, Minglong Shao, Taowei Yang, Meijuan Xu, Xian Zhang, Zhiming Rao

**Affiliations:** Key Laboratory of Industrial Biotechnology of the Ministry of Education, School of Biotechnology, Jiangnan University, Wuxi, 214122 Jiangsu China

**Keywords:** D-allulose 3-epimerase isomerase, Synergistic expression strategy, Auto-inducible promoter engineering, Acidic conditions

## Abstract

**Background:**

D-allulose, a hexulose monosaccharide with low calorie content and high sweetness, is commonly used as a functional sugar in food and nutrition. However, enzyme preparation of D-allulose from D-frutose was severely hindered by the non-enzymatic browning under alkaline and high-temperature, and the unnecessary by-products further increased the difficulties in separation and extraction for industrial applications. Here, to address the above issue during the production process, a tandem D-allulose 3-epimerase (DPEases) isomerase synergistic expression strategy and an auto-inducible promoter engineering were levered in *Bacillus subtilis* 168 (*Bs*168) for efficient synthesis of D-allulose under the acidic conditions without browning.

**Results:**

First, based on the dicistron expression system, two DPEases with complementary functional characteristics from *Dorea* sp. CAG:317 (*DS*dpe) and *Clostridium cellulolyticum* H10 (*RC*dpe) were expressed in tandem under the promoter HpaII in one cell. A better potential strain *Bs*168/pMA5-*DS*dpe-*RC*dpe increases enzyme activity to 18.9 U/mL at acidic conditions (pH 6.5), much higher than 17.2 and 16.7 U/mL of *Bs*168/pMA5-*DS*dpe and *Bs*168/pMA5-*RC*dpe, respectively. Subsequently, six recombinant strains based on four constitutive promoters were constructed in variable expression cassettes for improving the expression level of protein. Among those engineered strains, *Bs168*/pMA5-P_spoVG_-*DS*dpe-P_srfA_-*RC*dpe exhibited the highest enzyme activity with 480.1 U/mL on fed-batch fermentation process in a 5 L fermenter at pH 6.5, about 2.1-times higher than the 228.5 U/mL of flask fermentation. Finally, the maximum yield of D-allulose reached as high as 163.5 g/L at the fructose concentration (50% w/v) by whole-cell biocatalyst.

**Conclusion:**

In this work, the engineered recombinant strain *Bs168*/pMA5-P_spoVG_-*DS*dpe-P_srfA_-*RC*dpe was demonstrated as an effective microbial cell factory for the high-efficient synthesis of D-allulose without browning under acidic conditions. Based on the perspectives from this research, this strategy presented here also made it possible to meet the requirements of the industrial hyper-production of other rare sugars under more acidic conditions in theory.

## Background

D-allulose (D-psicose), the C-3 epimer of D-fructose, is a rare sugar with specialized health function [1]. Its sweetness is 70% of sucrose, but it contains ultra-low calories and is not easily absorbed by the digestive tract [2]. Currently, D-allulose has become an increasingly important functional biomolecule in food, dietary supplements, and pharmaceutical preparations and has been officially approved as a safe substitute for sucrose by the United States Food and Drug Administration (FDA) in 2014 [3, 4]. In addition, D-allulose possesses the characteristic of prebiotic functions and is beneficial in preventing or treating obesity and obesity-related inflammation [5, 6], regulating cholesterol metabolism [7], and protecting intestinal microbiota [8]. Consequently, the ingredients and functions of food naturally become the focus of research, and allulose has received widespread attention in this field.

Since Ken Izumori discovered the D-tagatose 3-epimerases (DTEases) and successfully realized the enzymatic preparation of D-allulose, the biosynthesis of D-allulose has entered the research stage [9, 10]. Because DTEases showed the highest substrate specificity towards D-tagatose, thus mass production of D-allulose from D-fructose using DTEase still remains a challenge [11]. Presently, biological synthesis of D-allulose is mainly achieved by D-fructose isomerization using D-allulose 3-epimerases (DPEases) [12], and the DPEases from multiple gene sources have been identified and reported (Table 1). However, most ever-reported enzymes exhibit optimum activity at alkaline and high temperature, which cause the non-enzymatic browning of sugars to be accelerated rapidly and generate unnecessary by-products under the reaction conditions, thereby leading to low purity and further burdening the downstream process. Therefore, an alternative method to solve the adverse impact on D-allulose production should be further investigated.

**Table 1:** DPEase characteristics of different sources

sources	Optimal pH	Optimal temperature(°C)	Half-life (At 60 °C)	Metal ion	Highest specificity	D-allulose/ D-fructose equilibrium ratio	References
*Clostridium cellulolyticum* H10	8.0	55	6.8 h	Co^2+^	D-allulose	32:68 (55 °C)	[25]
*Desmospora* sp. 8437	7.5	60	2.0 h	Co^2 +^	D-allulose	30:70 (60 °C)	[34]
*Dorea* sp. CAG317	6.0	70	1.0 h	Co^2 +^	D-allulose	30:70 (70 ℃)	[26]
*Clostridium scindens* ATCC 35,704	7.5	60	1.8 h	Mn^2+^	D-allulose	28:72 (50 °C)	[35]
*Clostridium bolteae* ATCC BAA-613	7.0	55	1.0 h	Co^2 +^	D-allulose	31:69 (55 °C)	[32]
*Ruminococcus* sp.	7.5–8.0	60	1.6 h	Mn^2+^	D-allulose	28:72 (60 °C)	[36]
*Agrobacterium tumefaciens*	8.0	50	3.99 min	Mn^2+^	D-allulose	33:67 (50 °C)	[37]
*Clostridium* sp.	8.0	65	15 min	Co^2 +^	D-allulose	28:72 (65 °C)	[38]
*Paenibacillus senegalensis*	8.0	55	140 min	Co^2+^ /Mn^2+^	D-allulose	30:70 (60 °C)	[14]
*Treponema primitia* ZAS-1	8.0	70	≤ 30 min	Co^2+^	D-allulose	28:72 (70 ℃)	[39]

Although the conventional mutation method can alter the physical and chemical properties of enzymes at the molecular level, significant developments are required, such as high-throughput screening conditions that are currently only suitable for some enzymes and not for DPEases activity detection [13]. In contrast, combinatorial expression of isoenzymes in one cell has been proven to be an excellent method to relieve the limitation of single enzyme-catalyzed reaction conditions [14, 15]. This was because isozymes could catalyze the same reactions, despite obvious differences in their molecular structure, physical and chemical properties, catalytic performance, and gene sequence. Thus, the expression of different DPEases in tandem may provide a clear guide for broadening the enzymatic properties and enhancing the synergistic interactions between isoenzymes.

In addition, according to the type of promoters in the genetic cassette of *B. subtilis*, heterologous protein production could be divided into constitutive expression and induced expression [16, 17]. However, induced expression requires expensive inducers (IPTG, maltose, etc.) and suitable induction conditions, limiting their practical application potential in expanded production [18]. Compared with inducible promoters, constitutive promoters do not need to be stimulated by adding exogenous inducers to produce target proteins during the culture process, thereby reducing production costs [19]. However, the strength of the promoter is closely related to the sequence structure of the target gene, and only the appropriate promoter can ensure a high yield of protein expression [15]. To date, several endogenous constitutive promoters are known in *B. subtilis*, such as P_HpaII_, P_43_, P_srfA_, and P_spovG_ [20−22]. But most researchers are accustomed to using the same promoter to express different genes, which results in the low production of target protein expression [23, 24]. Therefore, auto-inducible promoter engineering is necessary to develop various cassettes to enhance protein yields and improve specific enzyme activity.

In the present study, two DPEase isoenzymes from different genetic sources with complementary functional characteristics were combinatorially expressed in *Bs*168 to construct a food-grade recombinant strain with high catalytic efficiency and acid resistance. To further improve enzyme activity, six expression cassettes containing different native constitutive promoters were constructed. Finally, we successfully obtained a high specific enzyme activity strain that does not require additional inducers during the fermentation process.

## Results

### A non-enzymatic reaction occurred under alkaline conditions during the synthesis of D-Allulose catalyzed by single enzyme

**Fig. 1:**
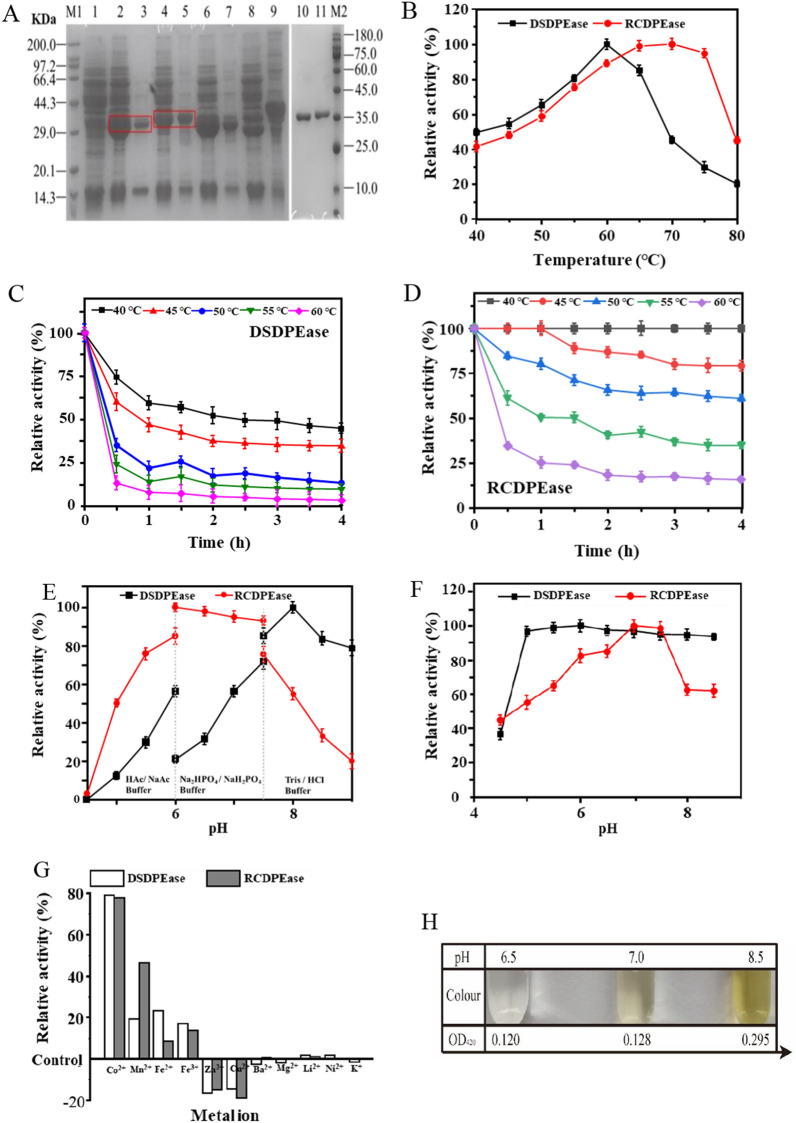
Enzymatic properties of purified DPEases and non-enzymic browning under alkaline conditions. **A** SDS-PAGE analysis. Lane 1: Control; lane 2: S_DS_ supernatant; lane 3: S_DS_ precipitation; lane 4: S_RC_ supernatant; lane 5: S_RC_ precipitation; lane 6: S_DS-RC_ supernatant; lane 7: S_DS-RC_ precipitation; lane 8: S_RC-DS_ Supernatant; lane 9: S_RC-DS_ precipitation; lane 10: purified DSDPEase; lane 11 purified RCDPEase. The target proteins were marked by a red box. **B** Effects of temperature on the activity of DEPases. **C** The thermostability of DEPases with 0.1 mM Co^2+^. **D** Effects of pH on the activity of DEPases. **E** The pH stability of DPEases with 0.1 mM Co^2+^. **F** Effects of metal ions on enzyme activity of DPEases. **G** The effects of pH on the browning of the reaction solution

*DS*dpe and *RC*dpe were expressed individually in *E. coli* had been investigated in our previous research. However, DPEases were expressed as an inclusion body, only a small amount of soluble protein can be detected in the supernatant (Data no shown). Considering the food safety requirements, *B. subtilis* 168 was chosen as chassis cells for heterologous expression of DPEases in the subsequent study. As shown in Fig. 1A, *DS*dpe and *RC*dpe were both expressed in a soluble form in *B. subtilis*, consistent with the relative theoretical molecular weight at approximately 33 kDa. And then, the effect of pH and temperature on the activity and stability of the purified enzymes were characterized separately. From the influence of temperature on the reaction, the activity of DSDPEase was significantly affected by temperature changes, reaching a peak at 60 ℃and dropping sharply at higher temperatures to below 80% (Fig. 1B). In contrast, RCDPEase activity was maintained above 80% in the range of 60–75 ℃ (Fig. 1B) and also exhibited better thermostability (Fig. 1D) than that did DSDPEase (Fig. 1C). However, from the influence of pH on the reaction, the activity of RCDPEase was significantly affected by changes in pH, whereas the activity of DSDPEase was less affected (Fig. 1E, F). The highest enzyme activity of RCDPEase under pH 8.5 reaction conditions is similar to the results of reported research [25]. And the activity of DSDPEase peaked at pH 6.0, which was consistent with the reported results [26]. In addition, different metal ions have varying effects on enzyme activity, and DSDPEase and RCDPEase showed maximum epimerization in the presence of Co^2+^ (Fig. 1G).

However, when the DPEases were used to catalyze the synthesis of D-allulose from fructose, the non-enzymatic browning of reaction mixture was found. As was shown in Fig. 1H, the reaction mixture was colorless (OD_420_ = 0.120) and transparent at pH 6.5, whereas it began to turn light yellow (OD_420_ = 0.128) at pH 7.0 and became dark yellow (OD_420_ = 0.295) at pH 8.0. This means that the phenomenon becomes more and more obvious as the pH of the reaction gradually becomes alkaline. Thus, inhibiting the non-enzymatic browning in the biological preparation of allulose has attracted our attention.

### Combinatorial expression of *DS*dpe and *RC*dpe in a Single Cell

**Fig. 2:**
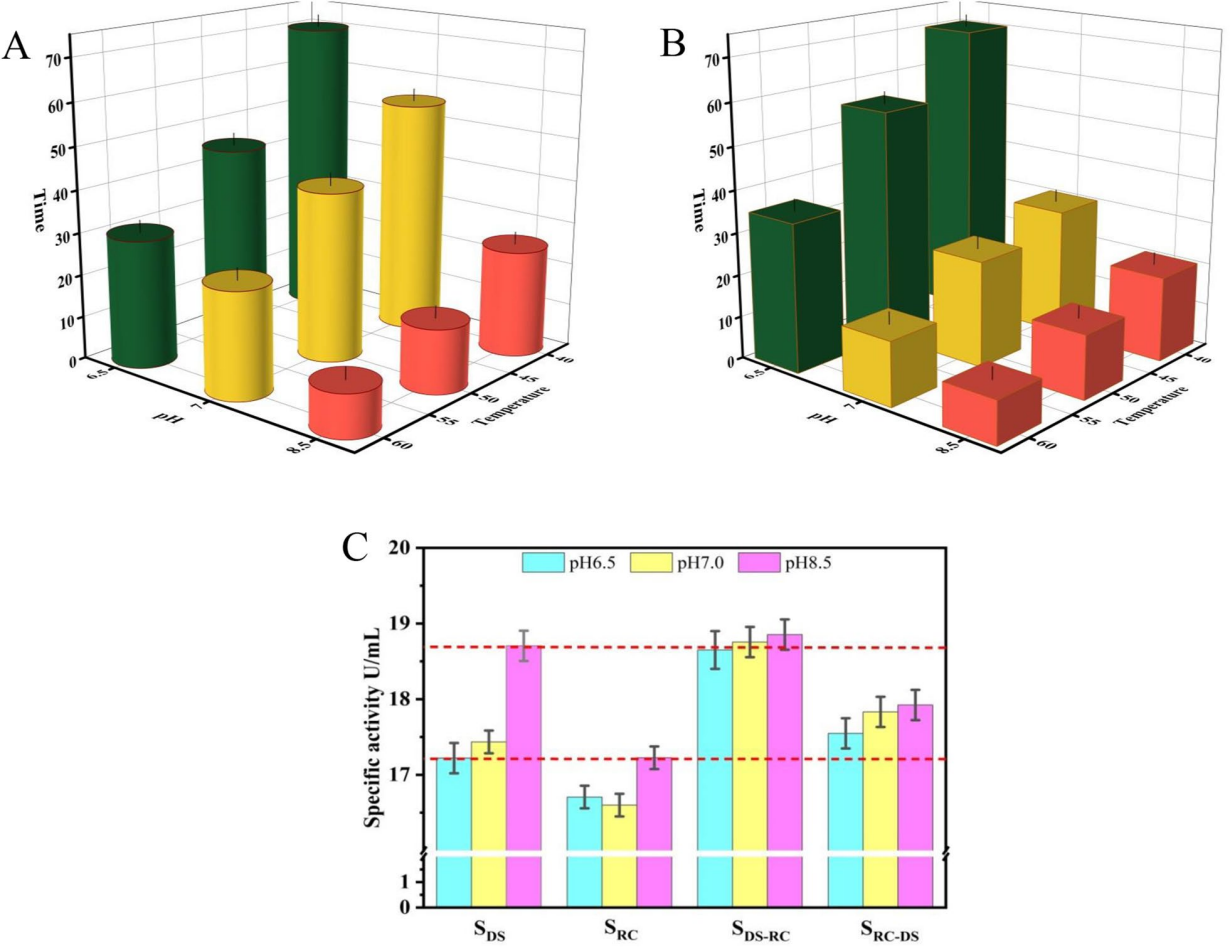
The equilibrium time for synthesis of D-allulose by purified DPEases and co-expression of DPEases in one cell. The time required for **A** DSDPEase and **B** RCDPEase to reach reaction equilibrium under different conditions. 100 g/L D-fructose was used as the substrate to synthesize D-allulose. **C** Enzyme activity of strains S_DS_, S_RC_, S_DS-RC_, S_RC-DS_

Studies have shown that the temperature and pH of the reaction solution are the key factors affecting non-enzymatic browning [27]. However, a relatively higher temperature is beneficial to reduce the viscosity of the sugar solution, accelerate the reaction, and improve the equilibrium conversion rate during the biotransformation process. Therefore, pH was used as one of the most critical influencing factors to eliminate the non-enzymatic browning reaction in this study. Unfortunately, the activity of a single enzyme under acidic conditions is considerably lower than that under alkaline conditions (Fig. 2A, B). Considering the complementary enzyme properties of DSDPEase and RCDPEase, the synergistic interactions between the two different DPEases were investigated.

To test the catalytic activity of combinatorial expression of DPEases in one cell, *Bs*168/pMA5-*DS*dpe-*RC*dpe and *Bs*168/pMA5-*RC*dpe-*DS*dpe were first constructed through the bicistronic vector system. As shown in Fig. 2C, the maximum enzyme activity of combinatorial expression strains was the same as that of the strain expressing a single DPEase. The optimum pH is all at 8.5. However, *Bs*168/pMA5-*DS*dpe-*RC*dpe exhibited the highest enzyme activity under different pH reaction conditions. Especially under acid conditions, the enzyme activity of *Bs*168/pMA5-*DS*dpe-*RC*dpe was 18.9 U/mL at pH 6.5, whereas the activity of *Bs*168/pMA5-*DS*dpe and *Bs*168/pMA5-*RC*dpe was only 17.2 U/mL and 16.7 U/mL, respectively. Therefore, this result indicates that combinatorial expression of DPEases in one cell could improve catalytic efficiency, and the non-enzymatic browning could be relieved by changing the pH of the reaction. However, there was still a certain gap between the enzyme activity under the weak acid range (around pH 6.5) and pH 8.5. The enzyme activity under weak acid conditions (pH 6.5) should need to be further enhanced.

### Improving specific enzyme activity of DPEases by an auto-inducible promoter engineering

**Fig. 3:**
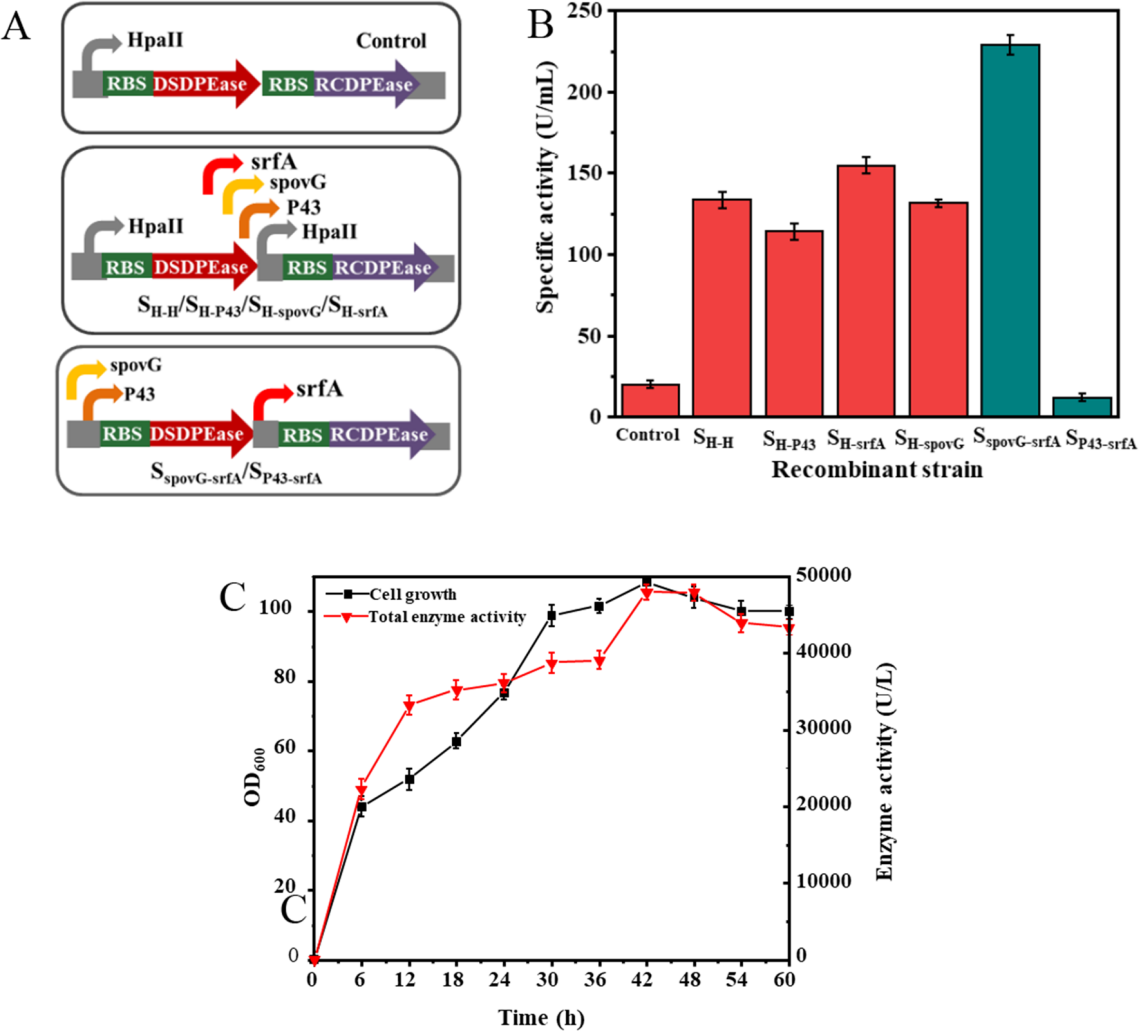
The effect of expression cassettes on enzyme activity. **A** The schematic diagram of promoter engineering in *B. subtilis*. **B** Comparison of crude enzyme activity of recombinant strains in flask fermentation. The transcription element has only one promoter, HpaII, as the original control. **C** Fed-batch fermentation of strain S_spovG-srfA_ was performed to test total enzyme activity in a 5 L bioreactor

To improve the expression level of DPEases in one cell, we attempted to optimize the promoter via an auto-inducible promoter engineering. First, four auto-inducible promoters (HpaII, P_43_, srfA, spovG) were inserted into the front of the *RC*dpe gene to construct the dual promoter vector pMA5-*DS*dpe-P_HpaII_-*RC*dpe, pMA5-*DS*dpe-P_43_-*RC*dpe, pMA5-*DS*dpe-P_srfA_-*RC*dpe, pMA5-*DS*dpe-P_spovG_-*RC*dpe, and then those plasmids were then transferred into *Bs168* to test their catalytic activity, the corresponding strains are abbreviated as S_H-H_, S_H-P43_, S_H-srfA_, and S_H-spovG_ (Fig. 3A). As shown in Fig. 3B, S_H-srfA_ showed the highest enzyme activity, the crude enzyme activities of S_H-srfA_ was 154.4 U/mL at pH 6.5, and also the enzyme activities of the S_H-H_, S_H-P43_, and S_H-spovG_ were increased to 133.3 U/mL, 114.0 U/mL, and 131.3 U/mL, respectively. This result proves that promoter optimization could promote the expression of DPEases. And then, pMA5-*DS*dpe-P_srfA_-*RC*dpe was used as the template, the original promoter HpaII of pMA5 was replaced with promoters P43 and spovG to construct S_P43-srfA_, S_spovG-psrfA._ In these two strains, the enzyme activity of S_spovG-psrfA_ was increased to 228.5 U/mL at pH 6.5, which was 148% that of S_H-srfA_ enzyme activity, and was 12.0–13.5 times that of the individual enzymes. However, the enzyme activity of S_P43-srfA_ was reduced to 11.6 U/mL, which might be caused by the mutual influence of transcription between the two different promoters.

Different promoters have different expression mechanisms in *B. subtilis*, mainly controlled by multiple factors such as growth time, environment, and nutrition. Thus, the fed-batch culture of strain S_spovG-srfA_ was performed to test total enzyme activity in a 5 L bioreactor. The rotating speed is coupled with DO during the fermentation process to ensure that the DO is controlled at 30%. As shown in Fig. 3C, the biomass increases rapidly in initially 6 h, whereas the pH value began to rise due to the consumption of sucrose, and then the feeding medium was continually supplemented to the bioreactor at this time. The cell density increased at the rate of 2–3 OD_600_ /h soon after the feeding, and the cell density of OD_600_ increased to 98.8, and the enzyme activity was 387.7 U/mL at 30 h. However, the growth of the recombinant strains entered the stable phase, and the cell density no longer increases after 36 h, but the enzyme activity still keeps increasing. When the recombinant strains were cultured to 42 h, the highest enzyme activity reached 480.1 U/mL. This result was approximately 11-fold higher than the previous studies about recombinant *C. glutamicum* [14]. In addition, S_spovG-srfA_ strain does not require an inducer during the fermentation process.

### Application of whole-cell transformation method in D-allulose production

**Fig. 4:**
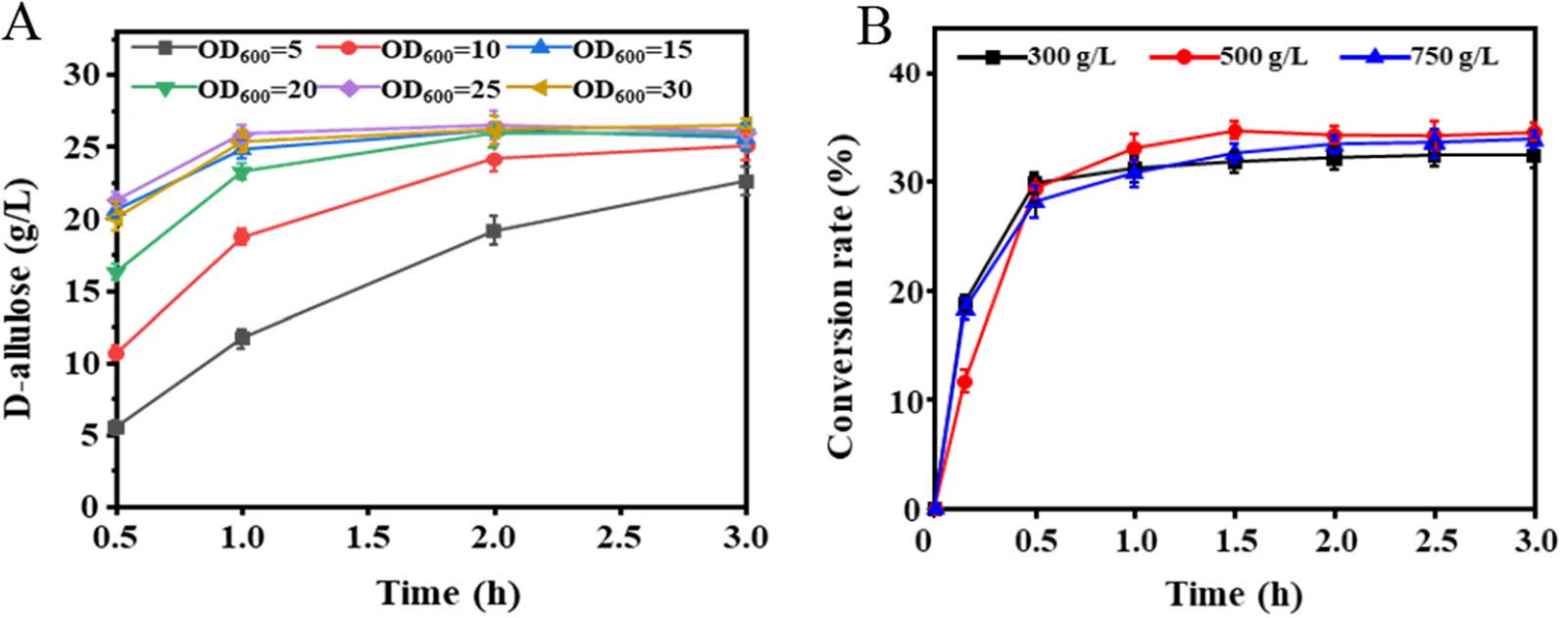
Optimization of reaction conditions for whole-cell biotransformation and recyclable synthesis of D-allulose by recombinant strain S_spovG-srfA_. **A** The effect of cell concentration OD_600_ = 5, 10, 15, 20, 25, 30 on the catalytic efficiency. **B** The effect of D-fructose concentrations on the catalytic efficiency

Compared with free enzyme catalysis, whole-cell transformation can eliminate the cumbersome steps of cell disruption and purification, and is more suitable for industrial production [28]. Therefore, we used resting cells of the strains S_spovG-srfA_ to prepare D-allulose from fructose via who-cell transformation at 60 °C and pH 6.5. Since cell density and substrate concentration are essential factors influencing biocatalysis, the catalysis process needs to be optimized. As was shown in Fig. 4A, the concentration of allulose increased with the increase of cell dosage in the first 1 h, while the yield of allulose at OD_600_ = 15 was not significantly different from others after 1.5 h. Considering the cost of culturing bacteria, the optimal cell dosage for whole-cell transformation was OD_600_ = 15. Moreover, the substrate concentration was related to the production efficiency of D-allulose, and a suitable substrate concentration could help maximize the yield. As was shown in Fig. 4B, when the concentration of fructose is 300 g/L, the equilibrium conversion rate of the substrate was 31.7%, which is lower than 32.7% and 32.6% of 500 g/L, 750 g/L, respectively. Although the equilibrium conversion rate at the substrate concentration of 500 g/L is the same as that of 750 g/L, too high substrate concentration will increase the viscosity of the conversion solution. Thus, 500 g/L D-fructose was the best substrate concentration for the synthesis of D-allulose. This result demonstrates that D-allulose could be synthesized efficiently without non-enzymatic browning, and the recombinant S_spovG-srfA_ exhibited great potential for D-allulose production at weak acidic conditions.

## Discussion

Under alkaline and high temperature, the hydroxyl groups of D-allulose could easily undergo the Maillard reaction with the amino groups, resulting in non-enzymatic browning, which affects the final product's appearance and generates more by-products [29]. Although browning is a widespread discoloration phenomenon in the food industry, removing non-enzymatic browning could effectively reduce the cost of downstream separation in industrial production.

Generally speaking, lowering the reaction temperature and pH are both effective ways to inhibit non-enzymatic browning in the biosynthesis of D-allulose [30, 31]. However, regardless of changing the temperature or pH, the synthesis efficiency of D-allulose will be significantly reduced. For example, Jia Min reported a neutral DPEase from *Clostridium bolteae* through screening different sources of genes, but the thermal stability of DPEase was poor at the optimum temperature [32]. Ben HlimaHajer also obtained a single mutant of glucose isomerase through molecular modification, the optimum pH was changed from 6.5 to 6.0, but its thermostability was significantly reduced [33]. Thus, an important problem for D-allulose production was balancing the high activity between the high temperature and low pH.

Considering the higher operating temperature required for industrial production of D-allulose, producing allulose at low pH was attempted in this study. However, the lack of new acid-resistant DPEases and the lower enzyme activity of the reported DPEases have restricted the production of D-allulose under acidic conditions. To obtain an acid-resistant strain, a tandem isomerase synergistic expression strategy was investigated. First, *DS*dpe and *RC*dpe were co-expressed with different expression orders in *B. subtilis*. As a result, the co-expressed strain S_DS-RC_ exhibited higher enzyme activity than the S_RC_ and S_DS_. This result has proven that the above strategy could compensate for the biochemical properties of single gene expression to a certain extent. However, the catalytic activity of enzymes is lower than alkaline conditions. To further improve the catalytic activity in a weak acidic environment and reduce the cost of production, an auto-inducible promoter engineering was optimized for increasing the expression of DPEases. Finally, the engineered strain *Bs168*/pMA5-P_spoVG_-*DS*dpe-P_srfA_-*RC*dpe exhibited the highest enzyme activity than the ever-reported. Even so, the current works still could be strengthened. We could try to express multiple genes in tandem from more sources in one cell than just the currently studied. In addition, mining strong constitutive promoters or modifying promoters can also be explored to improve enzyme expression levels in the future further.

## Conclusion

In summary, synthesize efficiently of D-allulose at high temperature and acidic pH without non-enzymatic browning was proved in this study. We first used a tandem DPEases isomerase synergistic expression strategy to overcome the insufficient activity of the single enzyme in one cell under acidic pH conditions. To further increase protein expression level and reduce the cost of protein expression, a recombinant strain *Bs168*/pMA5-P_spoVG_-*DS*dpe-P_srfA_-*RC*dpe with high performance was obtained through an auto-inducible promoter engineering. Finally, the engineered strain was successfully used for the biosynthesis of D-allulose in the whole-cell catalysis process without melanin formation. Thus, this strategy also provides a reference for constructing efficient microbial cell factories for the biosynthesis of other rare sugars under acidic conditions.

## Methods

### Bacterial strains and culture conditions

*E. coli* JM109 was used for cloning and plasmid preparation, *B. subtilis* 168 was used as a heterologous expression host. All bacterial strains and plasmids used in this study are shown in Table 2. The *E. coli* JM109 strain was grown in Luria–Bertani (LB) medium (containing 5 g/L yeast extract, 10 g/L tryptone, and 10 g/L NaCl) at 37 ℃ and 180 rpm. For enzyme production, *B. subtilis* 168 recombinant strains were first grown in 10 mL of LB medium containing 50 μg/mL kanamycin at 37 ℃ for 10–12 h and then transferred to TB medium (12 g/L tryptone, 24 g/L yeast extract, 5 g/L glycerol, 12.54 g/L K_2_HPO_4_, and 2.31 g/L KH_2_PO_4_). The strains were cultured at 37 ℃ and 180 rpm for 48 h in shake flasks.

**Table 2:** Strains, plasmids, and primers used in this study

Strains/plasmids/primers	Relevant genotype or phenotype	Sources
Strains		
*E. coli* JM109	*recA1*, *endA1*, *gyrA96*, *thi*^−1^, hsd R17(rk − mk +), supE44	Invitrogen
*E.coli JM109*/pMA5-*DS*dpe	*E. coli* JM109 containing pMA5-*DS*dpe (Amp^R^)	This study
*E. coli JM109*/pMA5-*RC*dpe	*E. coli* JM109 containing pMA5-*RC*dpe (Amp^R^)	This study
*B. subtilis* 168	Wild type	Laboratory stock
S_DS_	*B. subtilis* 168 containing plasmid pMA5-*DS*dpe (Km^R^)	This study
S_RC_	*B. subtilis* 168 containing plasmid pMA5-*RC*dpe (Km^R^)	This study
S_DS-RC_	*B. subtilis* 168 containing plasmid pMA5-*DS*dpe-*RC*dpe (Km^R^)	This study
S_RC-DS_	*B. subtilis* 168 containing plasmid pMA5-*RC*dpe-*DS*dpe (Km^R^)	This study
S_H-H_	*B. subtilis* 168 containing plasmid pMA5-*DS*dpe-P_HpaII_-*RC*dpe (Km^R^)	This study
S_H-P43_	*B. subtilis* 168 containing plasmid pMA5-*DS*dpe-P_43_-*RC*dpe (Km^R^)	This study
S_H-spovG_	*B. subtilis* 168 containing plasmid pMA5-*DS*dpe-P_spovG_-*RC*dpe (Km^R^)	This study
S_H-srfA_	*B. subtilis* 168 containing plasmid pMA5-*DS*dpe-P_srfA_-*RC*dpe (Km^R^)	This study
S_P43-srfA_	*B. subtilis* 168 *containin*g plasmid pMA5-P_43_-*DS*dpe-P_srfA_-*RC*dpe (Km^R^)	This study
S_spovG-srfA_	*B. subtilis* 168 containing plasmid pMA5-P_spovG_-*DS*dpe-P_srfA_-*RC*dpe (Km^R^)	This study
Plasmids		
pMA5	HpaII promoter, colE1, rep B, replicates in *E. coli* (Amp^R^) or *B. subtilis* (Km^R^)	Laboratory stock
pMA5-*DS*dpe	pMA5 derivative carrying gene *DS*dpe	This study
pMA5-*RC*dpe	pMA5 derivative carrying gene *RC*dpe	This study
pMA5-*DS*dpe-*RC*dpe	pMA5 derivative carrying gene *DS*dpe and *RC*dpe, sharing HpaII promoter	This study
pMA5-*RC*dpe-*DS*dpe	pMA5 derivative carrying gene *RC*dpe and *DS*dpe, sharing HpaII promoter	This study
pMA5-*DS*dpe-P_HpaII_-*RC*dpe	pMA5-*DS*dpe-*RC*dpe derivative carrying gene *DS*dpe、promoter HpaII and gene *RC*dpe	This study
pMA5-*DS*dpe-P_43_-*RC*dpe	pMA5-*DS*dpe-*RC*dpe derivative carrying gene *DS*dpe、promoter P_43_ and gene *RC*dpe	This study
pMA5-*DS*dpe-P_spovG_-*RC*dpe	pMA5-*DS*dpe-*RC*dpe derivative carrying gene *DS*dpe、promoter spovG and gene *RC*dpe	This study
pMA5-*DS*dpe-P_srfA_-*RC*dpe	pMA5-*DS*dpe-*RC*dpe derivative carrying gene *DS*dpe、promoter srfA and gene *RC*dpe	This study
pMA5-P_43_-*DS*dpe-P_srfA_-*RC*dpe	pMA5-*DS*dpe-P_srfA_-*RC*dpe derivative promoter HpaII replaced by the promoter P43	This study
pMA5-P_spovG_-*DS*dpe-P_srfA_-*RC*dpe	pMA5-*DS*dpe-P_srfA_-*RC*dpe derivative promoter HpaII replaced by the promoter spovG	This study
primers		
P1	5'-AAGTGAAATCAGGGGATCATGAACACGGAACGGAATTACTGCCTATTG -3'	This study
P2	5’-TTTCGACCTAGAGAACGCGTTTAGGTGGTGGTGGGTGGGTGGTTTCCATCCACACATATTTTCTGGAAATGCAAC -3’	This study
P3	5'-AAGTGAATCAGGGGGGGATGATGATAGAAACATGGTATACTACGCATTGG -3'	This study
P4	5'-TTTCGACCTCAGAAACGCGTTTAGGTGGTGGTGGGTGGGTGGGTGGGTGGGTGGTGGTGTTGATGATGATTCAATACTGGAGA -3'	This study

### Plasmid construction

The shuttle plasmid pMA5 of *E. coli*/*B. subtilis* was used for cloning and expressing protein. The original sequences of *DS*dpe (GenBank accession number: CDD07088.1) and *RC*dpe (GenBank accession number: ACL75304.1) without codon-optimized were synthesized at GENEWIZ in Suzhou, China. *DS*dpe and *RC*dpe with 6-histidine tag at the 3' end were inserted into the *Bam*HI and *Mlu*I restriction sites for constructing plasmids pMA5-*DS*dpe and pMA5-*RC*dpe, respectively. The 6-histidine tag was added at the 3' end of *DS*dpe and *RC*dpe by primers P1, P2, and P3, P4. *DS*dpe and *RC*dpe were fused by overlap extension PCR to construct bicistronic plasmids pMA5-*DS*dpe-*RC*dpe and pMA5-*RC*dpe-*DS*dpe. After the optimal expression orders were determined, the natural strong constitutive promoter was selected to improve the protein expression level further. HpaII promoter was cloned using the pMA5 plasmid as a template, and other promoters (P_43_, P_srfA,_ and P_spovG_) were cloned using the *B. subtilis* 168 genome as a template. Then recombinant plasmids pMA5-*DS*dpe-P_HpaII_-*RC*dpe, pMA5-*DS*dpe-P_43_-*RC*dpe, pMA5-*DS*dpe-P_spovG_-*RC*dpe, and pMA5-*DS*dpe-P_srfA_-*RC*dpe were constructed by fusing these promoters to the 5' end of the *RC*dpe fragment, respectively. Based on the optimization of second gene expression, the original HpaII promoter of pMA5 plasmid was replaced with promoters P_43_ and spovG. The expression levels of *DS*dpe and *RC*dpe were further increased by combining the promoters to construct pMA5-P_43_-*DS*dpe-P_srfA_-*RC*dpe and pMA5-P_spovG_-*DS*dpe-P_srfA_-*RC*dpe plasmids (Table 2).

### Expression and purification of DPEases

A single colony was selected and inoculated in 10 mL of LB medium, cultured for 10–12 h, and then transferred to the TB medium with 1% of the inoculum, cultured at 220 rpm for 20–24 h, centrifuged at 8,000 × g for 5 min to obtain the cells. The supernatant was discarded, and the collected cells were washed thrice with phosphate buffer (pH 7.0). The final total volume of the suspended cells was concentrated 10 times. Then, 25 μL of lysozyme (200 mg/mL) was added to 50 mL of the cell suspension, and the bacterial solution was overturned at regular intervals overnight at 4 °C. Cells were disrupted using an ultrasonic cell disruptor for 30 min. The supernatant obtained after centrifugation was used as the crude enzyme solution. The crude enzyme solution was first filtered using a 0.22 μm filter before loading onto a 1 mL Ni affinity column (GE Healthcare, HisTrap HP) that was pre-equilibrated with 50 mM equilibration buffer (20 mM Tris and 500 mM NaCl, pH 7.4). Then purified DPEases were collected using elution buffer (20 mM Tris, 500 mM NaCl and 500 mM imidazole, pH 7.4) with a stepwise linear gradient. The purified protein was analyzed by Sodium dodecyl sulfate–polyacrylamide gel electrophoresis (SDS-PAGE). The concentration of protein was determined through the Bradford method. The absorbance value of the BSA standard solution and protein sample was measured at 595 nm using a microplate reader, BioTek Instruments Ocean International, Beijing, China.

### Enzyme assays

DSDPE and RCDPE were assayed for their D-allulose-forming activity using 555 mM fructose as substrate in 50 mM Na_2_HPO_4_/ NaH_2_PO_4_ buffer (pH 6.0). Specifically, the reaction mixture consisting of 900 μL of 616 mM sucrose solution, 100 μmol Co^2+^ and 100 μL of purified enzymes in a final volume of 1 mL. The enzyme activity of DSDPE and RCDPE was measured at 55 °C, 70 °C for 10 min, respectively. The reaction was terminated by boiling the samples at 100 ℃ for 10 min. The allulose production was quantified by high-performance liquid chromatography (HPLC). One unit (U) of enzyme activity was defined as the amount of enzyme that catalyzed the formation of 1 μmol D-allulose in 1 min under the described conditions.

### Determination of temperature and pH on enzyme activity

Effects of temperature on the activity of DEPases were determined between 40 °C and 80 °C in Na_2_HPO_4_/ NaH_2_PO_4_ buffer (pH 6.0). Thermal stability of DPEases were determined at 40 °C, 45 °C, 50 °C, 55 °C, 60 °C, respectively. Samples were taken every 30 min, and then the residual activity of DPEases after different incubation time was performed as described above. The original activity without incubation was taken as 100%.

The optimal pH value of enzyme activity was assayed at different values from 4.5–9.0 using acetic acid/sodium acetate buffer (pH 4.5–6.0), Na_2_HPO_4_/ NaH_2_PO_4_ buffer (pH 6.0–7.5), and Tris/HCl buffer (pH 7.5–9.0). The pH stability of DPEases was measured after storing pure enzyme in different buffers for 2 h at 4 ℃. In addition, the effects of metal ions on enzyme activity of DPEases were studied in the presence of 100 μmol various metal ions (Fe^2+^ 、Mn^2+^ 、Mg^2+^ 、Zn^2+^ 、Co^2+^、K^+^、Ba^2+^、Fe^3 +^、Cu^2+^ 、Ni^2+^ 、Li^2+^). EGTA was taken as a control. All the above assays were repeated three times.

### Preparation of DPEase by recombinant *B. subtilis* cells

*B. subtilis* cells were cultured in 100 ml TB medium at 37 °C for 24 h. The kanamycin with a concentration of 50 mg/L was supplemented into the culture to maintain the stability of the expression plasmid. Harvested cells were washed 3 times with PB buffer and centrifuged at 8,000 × g for 15 min. Concentrated bacterial suspension stored at − 40 °C after supplement of 10% glycerol (v/v).

Fed-batch fermentation of stain S_spovG-srfA_ was carried out in a 5 L bioreactor (DIBIER, Shanghai, China) containing 2 L fermentation medium (sucrose 15 g/L, yeast powder 20 g/L, Na_2_HPO_4_ 4 g/L, NaH_2_PO_4_ 1 g/L, NaCl 8 g/L). The 100 mL seed culture was first inoculated into bioreactor after cultivating 15 h at 37 °C, and then the pH of the culture medium was controlled at 7.0. After a period, the feeding medium (sucrose 300 g/L, yeast powder 70 g/L) (coupled with pH) was added into the bioreactor when the pH increased and continuous fermentation for 60 h. Samples at different fermentation times were collected and placed on ice.

### Determination of specific and total activities for D-allulose production by recombinant cells

The reaction mixture (1 mL final volume) for enzyme activity determination contained D-fructose (100 g/L), PB buffer (pH 6.5, 50 mmol/L NaH_2_PO_4_/Na_2_HPO_4_), and Co^2+^ (100 μmol/L), and the crude enzyme solution (200 μL) was added to initiate the reaction. The reaction was incubated at 60 °C for 2 min in a metal shaker and was then stopped by boiling for 10 min. Cells were washed three times with NaH_2_PO_4_/Na_2_HPO_4_ buffer (pH 7.0) and then suspended in 100 μL of the same buffer. In addition, the whole-cell mixture contained 900μL of 300 g/L D-fructose solution, 100 μmol/L Co^2+^. The reaction was continued for 5 min in a metal shaker and stopped by boiling for 10 min. The definition of unit enzyme activity was the same as the above described.

### Optimization of the conditions for whole-cell biotransformation

The optimal cell dosage was determined in a 30 mL reaction system at 60 °C, 200 rpm, and the final OD_600_ of the cell density is 5, 10, 15, 20, 25, and 30, respectively. Substrate solutions with fructose concentrations of 300, 500, and 750 g/L were investigated at the optimal cell dose for optimizing the substrate concentration, and the reaction was carried out under standard conditions. The yield of D-allulose was measured at 30 min intervals.

### Analytical methods

The cells' optical density (OD600) was measured at 600 nm using an ultraviolet–visible spectrophotometer (UV-1200), Aoxiang Instrument Co., Ltd., Shanghai, China. The content of D-fructose and D-allulose was determined by HPLC (Agilent Technologies, 1260 Infinity) with RID detector using Hi-Plex Ca column (300 mm × 7.7 mm), ultrapure water was used as the mobile phase with the flow rate of 0.4 mL/min at 80 °C. The browning measurement method of the reaction solution: the absorbance of different samples was measured at a wavelength of 420 nm.

## Data Availability

All data involved in this study, if not found in this article or supplementary material, may be obtained from the corresponding author.

## Abbreviations

DPEase: D-allulose 3-epimerase
*Bs*168: *Bacillus subtilis* 168
FDA: Food and Drug Administration
DTEases: D-tagatose 3-epimerases
